# The Influence of SARS-CoV-2 Infection on the Development of Selected Neurological Diseases

**DOI:** 10.3390/ijms25168715

**Published:** 2024-08-09

**Authors:** Klaudia Kryńska, Katarzyna Kuliś, Wiktoria Mazurek, Monika Gudowska-Sawczuk, Monika Zajkowska, Barbara Mroczko

**Affiliations:** 1 Department of Biochemical Diagnostics, Medical University of Bialystok, Waszyngtona 15A St., 15-269 Bialystok, Polandbarbara.mroczko@umb.edu.pl (B.M.); 2 Department of Neurodegeneration Diagnostics, Medical University of Bialystok, Waszyngtona 15A St., 15-269 Bialystok, Poland; monika.zajkowska@umb.edu.pl

**Keywords:** COVID-19, SARS-CoV-2, CNS, multiple sclerosis, ischemic stroke, Alzheimer’s disease, neuroinflammation

## Abstract

In 2024, over 775 million cases of COVID-19 were recorded, including approximately 7 million deaths, indicating its widespread and dangerous nature. The disease is caused by the SARS-CoV-2 virus, which can manifest a wide spectrum of symptoms, from mild infection to respiratory failure and even death. Neurological symptoms, such as headaches, confusion, and impaired consciousness, have also been reported in some COVID-19 patients. These observations suggest the potential of SARS-CoV-2 to invade the central nervous system and induce neuroinflammation during infection. This review specifically explores the relationship between SARS-CoV-2 infection and selected neurological diseases such as multiple sclerosis (MS), ischemic stroke (IS), and Alzheimer’s disease (AD). It has been observed that the SARS-CoV-2 virus increases the production of cytokines whose action can cause the destruction of the myelin sheaths of nerve cells. Subsequently, the body may synthesize autoantibodies that attack nerve cells, resulting in damage to the brain’s anatomical elements, potentially contributing to the onset of multiple sclerosis. Additionally, SARS-CoV-2 exacerbates inflammation, worsening the clinical condition in individuals already suffering from MS. Moreover, the secretion of pro-inflammatory cytokines may lead to an escalation in blood clot formation, which can result in thrombosis, obstructing blood flow to the brain and precipitating an ischemic stroke. AD is characterized by intense inflammation and heightened oxidative stress, both of which are exacerbated during SARS-CoV-2 infection. It has been observed that the SARS-CoV-2 demonstrates enhanced cell entry in the presence of both the ACE2 receptor, which is already elevated in AD and the ApoE ε4 allele. Consequently, the condition worsens and progresses more rapidly, increasing the mortality rate among AD patients. The above information underscores the numerous connections between SARS-CoV-2 infection and neurological diseases.

## 1. Introduction

Coronaviruses (CoV) are a group of viruses that cause mild to severe respiratory infections in humans [1]. The first records of human coronaviruses date back to the 1960s, but in December 2019, the first cases of pneumonia caused by the newly identified coronavirus were reported in Wuhan, China [2]. In early 2020, the World Health Organization (WHO) described this virus as severe acute respiratory syndrome coronavirus 2 (SARS-CoV-2), causing a life-threatening disease known as COVID-19. COVID-19 quickly spread around the world and on 11 March 2020, the disease was declared a global pandemic [3]. By May 2024, over 775 million cases of COVID-19 had been reported, including approximately 7 million deaths, making it one of the deadliest pandemics in history [4].

SARS-CoV-2 belongs to the *Betacoronavirus* genus of the *Coronaviridae* family, which comprises a large group of pathogens affecting both humans and animals. SARS-CoV-2 is a newly mutated RNA virus that primarily infects the respiratory and gastrointestinal systems, manifesting in a wide array of symptoms [5,6]. Observational studies by e.g., Pal et al. [6], Ramzy et al. [7], and Ousseiran et al. [8] have documented clinical manifestations in patients, including fever, dyspnea, loss of smell or taste, cough, or nausea. Although the course of the disease can be asymptomatic or mild, there are severe cases that lead to systemic inflammation, cardiopulmonary arrest, or even death. The risk of organ damage and fatal complications is significantly increased in patients with comorbidities such as i.e., diabetes, chronic obstructive pulmonary diseases, hypertension, or obesity [6,7,8]. It has been observed that SARS-CoV-2 infection may also involve the central nervous system (CNS). Neurons and neuroglial cells susceptible to viral infections are consequently exposed to damage caused by inflammation in the CNS [9]. The virus entering the CNS can cause changes both at the level of nerve cells and in the entire brain [10]. The hypothetical mechanism linking SARS-CoV-2 infection with neurological diseases includes, among others, abnormalities in brain blood vessels and damage to the vascular endothelium, hypercoagulability, ischemia, hypoxia, inflammasome activation or the so-called cytokine storm [11,12,13].

Taking the above into account, the aim of this literature review was to assess the impact of SARS-CoV-2 virus infection on the development of selected neurological disorders. Multiple sclerosis (MS), ischemic stroke (IS), and Alzheimer’s disease (AD) share common features which involve neuroinflammation and oxidative stress, which can lead to cognitive impairment. The course of these diseases is influenced by the condition of blood vessels, the blood-brain barrier (BBB), and several risk factors such as age or lifestyle. In all the above-mentioned disorders, viral infections can origin systemic and local inflammation, which is a common cause of AD (impact of HSV-1), multiple sclerosis (impact of Epstein-Barr virus), and ischemic stroke (impact of e.g., influenza viruses). Viral infections such as SARS-CoV-2 may affect the condition of the BBB and, therefore, influence the development of the aforementioned diseases. Multiple sclerosis, ischemic stroke, and Alzheimer’s disease are related to the occurrence of neurodegenerative processes and can be observed as a result of direct invasion of a virus via the olfactory or gustatory receptors, coagulopathy, generation of reactive oxygen species (ROS), induction of autoimmunity and chronic inflammation. All those processes can be induced by the SARS-CoV-2 virus, can impair immune system function and, as a result, influence the development of these diseases.

## 2. Multiple Sclerosis

MS is an inflammatory disease of CNS characterized by neurodegenerative changes. Its etiology remains partially unknown, but it affects approximately 2.3 million people worldwide, including young individuals [14,15]. The disease is influenced by genetic and environmental factors, such as Epstein–Barr virus (EBV) infection, reduced vitamin D levels, and cigarette smoking [16,17]. In MS, the white matter of the CNS is destroyed due to an autoimmune process [18]. There are two main hypotheses regarding the development of MS. The first autoimmune hypothesis is that autoreactive T cells move from the periphery to the brain through BBB, where they are activated by antigen-presenting cells (APCs), like dendritic cells, causing inflammation. Second, the neurodegenerative hypothesis suggests that MS is primarily a neurodegenerative disease that leads to an autoimmune reaction [17]. MS can manifest in three clinical forms: Relapsing-Remitting Multiple Sclerosis (RRMS; ~85% of MS patients), Primary Progressive Multiple Sclerosis (PPMS; ~10–20% of patients), and Secondary Progressive Multiple Sclerosis (SPMS) [19]. Symptoms of MS vary depending on the involvement of sensory, motor, visual, and brainstem pathways. Common symptoms include optic neuritis, transverse myelitis, brainstem syndrome, and cognitive impairments [20,21]. RRMS form features periods of neurological disorders alternating with remissions, with a recurrence rate of 1.5 episodes per year. Symptoms include sensory disturbances, balance issues, and vision problems. On the other hand, PPMS is characterized by the absence of an initial relapsing phase and continuous disease progression. SPMS often develops from RRMS, with relapses and periods of stable disability. The transition from RRMS to SPMS typically takes about 19 years. Factors like older age at RRMS onset and male gender increase the risk of transition [22].

Diagnosis relies on clinical symptoms, neurological examinations, cerebrospinal fluid analysis, and magnetic resonance imaging (MRI). The McDonald criteria are commonly used for diagnosing MS due to the lack of a specific test [20,23,24]. It is worth emphasizing that disease-modifying therapy (DMT) is most effective for RRMS, with options like interferon type β and monoclonal antibodies such as natalizumab and daclizumab. A complete cure is not yet achievable, and the disease may progress despite treatment [25].

Research indicates that EBV infection is a potential cause of MS, with other viruses possibly contributing [26]. Viral infections may lead to chronic inflammation, allowing immune cells to penetrate the CNS and cause further damage. This includes the infiltration of T lymphocytes through a damaged BBB and the release of viral products, which can stimulate an inflammatory response [27].

SARS-CoV-2 is a neuroinvasive virus. The virus enters the brain through the pericytes and astrocytes of the BBB. This occurs as a result of the expression of the ACE2 receptor triggered by the SARS-CoV-2 virus on the brain endothelial cells [28]. Protein S has properties in how it controls the continuity of the BBB and triggers effects on the function of this structure [28]. The presence of the ACE2 receptor in tissues determines the cellular tropism of the virus, which attacks the CNS, resulting in neuronal damage [29]. There, as a result of the excessive synthesis of neurotoxic cytokines such as interleukin (IL)-1β and IL-6, vascular and demyelinating changes occur. Research has shown that as a result of infection with the SARS-CoV-2 virus, glial cells produce chemokines. This leads to nerve destruction. A similar mechanism occurs in MS [30].

A factor involved in the progression of MS is also mitochondrial dysfunction which may be caused by SARS-CoV-2 infection. It has been revealed that SARS-CoV-2 proteins localize to mitochondria which leads to e.g., damage and mutations in mitochondrial DNA [31]. Malfunction of these structures leads to intracellular dysregulation and a decrease in energy production. A decrease in ATP production disrupts the transmission of electrical signals. As a result of mitochondrial dysfunction, neurons are damaged [31]. Going forward, it is well known that mitochondria are a significant source of ROS, which can damage cells. Munoz-Jurado A et al. discovered that excessive production of reactive oxygen species and reactive nitrogen species (RNS), which have oxidizing properties, stimulates nuclear factor kappa B (NF-κB) in nerve cells such as astrocytes, oligodendrocytes, and neurons. NF-κB affects the synthesis of MS development factors like TNF-α, nitric oxide, and IL-1α. This results in increased inflammation, demyelination, and the onset of the disease [32]. Moreover, the theoretical thesis suggests that molecular mimicry between self-antigens and viral antigens, along with a delayed immune response post-infection, are among the mechanisms linking COVID-19 with MS. Studies by Lima et al. [33] have indicated that individuals infected with SARS-CoV-2 and experiencing nervous system symptoms often exhibit autoantibodies against neurons or glial cells in their cerebrospinal fluid (CSF). This phenomenon contributes to the destruction of CNS structures and potentially impacts the onset of MS [33]. On the other hand, it has been suggested that B lymphocytes are involved in the pathogenesis of MS. However, this involvement is not related to the presence of antibodies but rather to an imbalance in the production of different cytokines. B cells synthesize excessive amounts of pro-inflammatory cytokines and insufficient amounts of regulatory cytokines (e.g., IL-35), resulting in an abnormal pro-inflammatory response from Th1, Th17, and myeloid cells. Cellular infiltrates consisting of B cells have been detected in people with MS and the concentration of B lymphocytes, plasma blasts, and plasma cells is increased in the cerebrospinal fluid of MS patients. In this study, the authors define the role of autoantibodies in the pathogenesis of MS as unknown [34]. Moreover, it is known that viral infections, including SARS-CoV-2, affect the production of interferons. Physiologically, they assist in suppressing viral replication and activating immune cells like macrophages and natural killer cells. However, it has been observed that in severe COVID-19 cases, IFN responses can be dysregulated [35]. Defective production of e.g., IFN-1 can lead to hyper inflammation aggravating the disease. Going further, in patients with MS, impairment of IFN-1 response at the genetic and transcriptional levels has been also reported [35]. Therefore, external interferons, particularly IFN-β, are employed in MS treatment to regulate the immune response and reduce the frequency and severity of relapses. Thus, knowing that SARS-CoV-2 infection can interfere with IFN pathways, it appears that the virus may act as an environmental trigger for MS in susceptible individuals, contributing to the onset of the disease in genetically predisposed individuals or potentially exacerbating symptoms [35,36].

COVID-19 and MS are linked to various immunological disturbances that might potentially influence each other’s progression. It has been observed that the SARS-CoV-2 virus causes a strong inflammatory response combined with autoimmunity and the release of pro-inflammatory cytokines, such as IL-17, which is produced primarily by Th17 cells. It is worth emphasizing that IL-17 is an important cytokine in the immune response to SARS-CoV-2, contributing to both virus removal and inflammation. However, enhanced synthesis of IL-17 results in, among other things, the production of coagulation factors and contributes to multi-organ failure. Unfortunately, it has been observed that IL-17 is also crucial in the development and progression of MS, as it drives the inflammatory and autoimmune responses that cause demyelination, nerve damage, and disruption of BBB. Therefore, information available in the literature suggests that SARS-CoV-2 infection may potentially exacerbate multiple sclerosis symptoms or influence disease progression through IL-17-mediated mechanisms [35,36].

Another crucial factor at the intersection of COVID-19 and MS is the inflammasome. It is a complex molecular platform that consists of inflammatory caspases, sensor protein, and sometimes an adapter protein that connects the other two components. Abnormal inflammasome activation can be linked to infections and inflammatory diseases. Inflammasomes, particularly NLR family pyrin domain containing 3 (NLRP3), have been significantly implicated in the development of MS. The NLRP3 plays a pivotal role in T cell polarization, BBB damage, and neurodegeneration. Moreover, the capacity of SARS-CoV-2 to activate this platform has been observed during more severe cases of COVID-19. So, a major factor in COVID-19 pathogenesis and its severity is probably the cytokine storm linked to NLRP3 overactivation. Thus, it appears that the progression of multiple sclerosis could be significantly influenced by SARS-CoV-2 infection [35,36]. An alternative theory suggests that the inflammasome, after forming a complex with the adapter protein ASC, activates caspase-1. This activation leads to the processing and activation of IL-1β, IL-18, and gasdermin, which subsequently cause pyroptosis, i.e., the death of nervous system cells. According to the authors, the consequence of these processes may not necessarily be cell death but rather a permanent dysregulation of the inflammasome, which has a considerable impact on the speed of disease onset [27].

Conversely, individuals with multiple sclerosis undergo treatments that modulate their immune systems, increasing susceptibility to viral infections [37]. The most prevalent drug used in MS treatment, rituximab, a monoclonal antibody, targets CD20 B cells to suppress the humoral response. However, despite its efficacy, rituximab poses a considerable risk of infection, rendering MS patients treated with this drug more vulnerable to severe COVID-19 [38]. Another medication that heightens the risk of COVID-19 is sphingosine-1-phosphate receptor modulators (S1PRM) [30].

**Figure 1:**
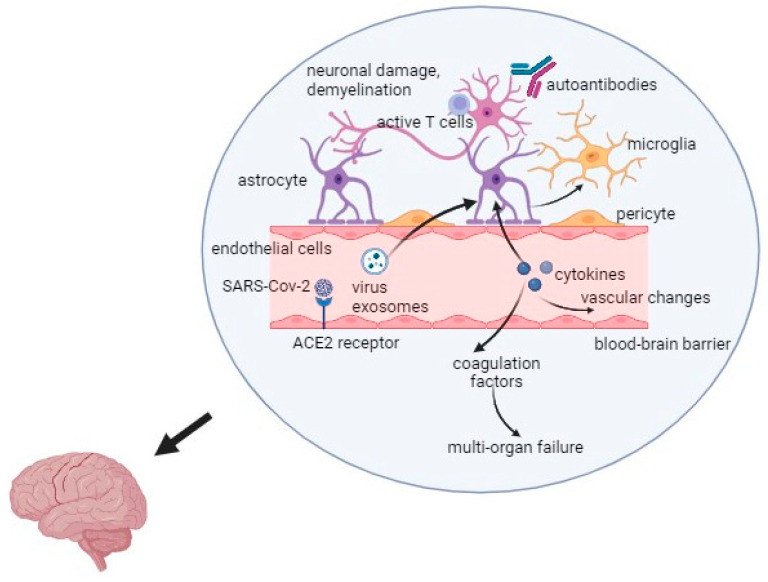
Mechanism of the SARS-CoV-2 infection in Multiple Sclerosis. Captions: ACE2—angiotensin converting enzyme 2; SARS-CoV-2—severe acute respiratory syndrome coronavirus 2.

Interestingly, the latest research results suggest that the development and relapses of multiple sclerosis may be influenced by stress related to the COVID-19 pandemic. Long-term stress affects the immune system functions and changes the secretion of dopamine in the mesolimbic system of the brain [39]. Hyperactivity of the hypothalamic-pituitary-adrenal axis and increased production of cortisol in response to stress may cause the progression of MS [39]. The overall mechanism of the virus action is presented in Figure 1.

To sum up, infection with the SARS-CoV-2 virus influences the development of multiple sclerosis. This is related to dysregulated immune pathways that are found in severe cases of COVID-19. Studies carried out by e.g., Fernandes de Souza et al. show the interaction of SARS-CoV-2 with genes for autoimmune diseases, including MS. The three main pathways are the IFN-1 response, the Th1/Th17 axis, and the inflammasome pathway. It is speculated that the negative effects of COVID-19 may be visible in the future [36]. What is more, it seems that SARS-CoV-2 has an impact on individuals with MS, particularly regarding the potential for disease exacerbation. SARS-CoV-2 can trigger an exaggerated immune response, leading to a cytokine storm. This hyperinflammatory state may exacerbate MS symptoms by enhancing existing inflammation in the CNS [30]. The virus may also influence T-cell activation and differentiation, potentially aggravating autoimmune processes in MS patients. SARS-CoV-2 infection has been shown to affect the integrity of the BBB, potentially allowing more immune cells to enter the CNS and worsen MS pathology [27]. In addition, many MS patients are on immunosuppressive therapies that may increase susceptibility to infections, including COVID-19. Some DMTs might alter the course of COVID-19 or affect the severity of MS relapses during the infection [25]. It is also worth noting that older age and advanced disease stages in MS patients are associated with a higher risk of severe COVID-19 outcomes and possible exacerbation of MS symptoms. However, ongoing research is essential to fully understand the relationship between COVID-19 and MS and to develop interventions that address both conditions.

## 3. Ischemic Stroke

Stroke, a cerebrovascular disorder that lasts at least 24 h, causes nerve cell damage due to decreased blood supply to the brain [40]. Strokes are categorized into ischemic and hemorrhagic types. Ischemic stroke occurs when a blood vessel is blocked, interrupting blood flow to brain tissue, while hemorrhagic stroke involves intracranial bleeding from a vessel rupture [40,41]. Approximately 60–80% of strokes are ischemic [41,42]. Stroke is a leading cause of mortality and disability globally [43]. Importantly, the global stroke burden is expected to rise in the next decade [44]. Ischemic stroke is influenced by non-modifiable and modifiable risk factors. Non-modifiable factors include age, gender, ethnicity, and genetics [45]. Although strokes can occur at any age, they predominantly affect older individuals [46,47]. However, 10–15% of cases occur in young adults aged 18 to 49 [48,49]. Women are generally more susceptible to ischemic stroke than men due to factors like longer life expectancy, hormonal contraception, and pregnancy [46,47,49]. Modifiable risk factors significantly influence stroke development, with hypertension being the most notable. High blood pressure, combined with lifestyle factors like smoking, alcohol consumption, substance abuse, and inactivity, as well as conditions such as diabetes, heart disease, hyperlipidemia, and obesity, significantly increases stroke risk [43,47,50].

Both viral and bacterial infections, particularly those affecting the respiratory tract, pose significant risk factors for cerebrovascular events [51,52]. Numerous clinical studies (i.a.Smeeth et al. [51] and Bahouth et al. [53]) have revealed an association between recent systemic infections and the occurrence of strokes. It is hypothesized that the primary pathomechanism involves the activation of immune processes in predisposed individuals with coagulation disorders or dysfunctions of the vascular endothelium [51,53].

Viruses may precipitate ischemic stroke through several interconnected mechanisms, with vasculopathy being a prominent factor. Systemic infection, persistent inflammation, and immune complex deposition can induce vasculitis, rupture of atherosclerotic plaques, and alteration of vascular wall structure. These factors contribute to vascular damage, increasing the risk of cerebrovascular events. Furthermore, inflammation associated with viral infections can foster the development of atherosclerotic lesions, adversely affecting blood flow in brain tissue. The destabilization and rupture of these atherosclerotic plaques significantly heighten the risk of ischemic stroke [53,54].

Another crucial pathomechanism associated with acute viral illness is the disruption of blood coagulation processes, manifesting as hypercoagulability and thrombosis. Viral infections may trigger the synthesis of acute phase proteins and initiate the coagulation cascade. Additionally, platelet function may be impaired, leading to increased activation and aggregation. These coagulation abnormalities contribute to the formation of blood clots and thromboembolism. The resultant narrowing or complete occlusion of blood vessels restricts blood flow to the brain, which can precipitate ischemic events [53,55,56]. These mechanisms highlight the complex interplay between viral infections and stroke risk, emphasizing the need for continued research to better understand these pathways and develop effective strategies for prevention and management.

A variety of viruses have been potentially linked to an increased risk of developing acute ischemic stroke [53,56]. One such virus is SARS-CoV-2. Since the onset of the global COVID-19 pandemic, numerous neurological complications arising from severe infection have been reported [57]. While common mild symptoms related to the nervous system include headache, dizziness, and impaired smell or taste, some patients may experience more severe cerebrovascular and neuropsychiatric manifestations [58]. Many researchers have noted an increased risk of stroke in individuals with COVID-19. Research indicates that the incidence is approximately 1–3% among hospitalized patients and 6% among those in intensive care units [59]. The majority of individuals with COVID-19 who experience a stroke are male (62%), with a mean age of 63 years [60]. Notably, most of these patients present crucial risk factors, such as hypertension, diabetes mellitus, or hyperlipidemia [60,61]. Studies carried out by Merkler et al. [62] suggest that cerebrovascular disorders are more prevalent in COVID-19 patients compared to those with influenza [62]. Although SARS-CoV-2 infection is not an independent cause of stroke, it plays a significant role in its development [60].

COVID-19 is a relatively newly discovered disease, and further research is needed to determine the precise causes of stroke in patients with SARS-CoV-2 infection. Nevertheless, there are several assumptions regarding the potential pathomechanisms of cerebrovascular complications. Coronaviruses, including SARS-CoV-2, enter cells via the angiotensin-converting enzyme 2 (ACE-2) receptor, predominantly expressed in the endothelium of vessels and internal organs, such as the brain [63,64]. The binding of the viral spike glycoprotein to receptors on infected cells facilitates the entry and release of viral genetic material [63]. SARS-CoV-2 contains a positive-sense single-stranded RNA, recognized by pattern recognition receptors (PRRs), primarily Toll-like receptors (TLR), initiating the host’s immune response [63,65]. The presence of ACE-2 receptors in the brain suggests that SARS-CoV-2 may directly affect the nervous system. Indirectly, the risk of ischemic stroke may increase due to the expression of these receptors in the myocardium and endothelium of vessels. The virus may disrupt cardiac muscle cell function, leading to hypoxia, arrhythmias, and thrombotic complications [64,66].

Moreover, hypoxia often occurs in COVID-19 patients. One possible mechanism is the blockade of iron transport caused by the interaction between hepcidin and the SARS-CoV-2 spike glycoprotein [67,68]. Hepcidin, a peptide hormone, plays a significant role in systemic iron homeostasis control [69]. Additionally, approximately 20% of the body’s total oxygen supply reaches the brain, making it highly sensitive to oxygen content changes, even slight deficiencies can cause irreversible changes in brain tissue [67]. Inadequate oxygen supply affects coagulation and fibrinolysis processes. Hypoxia-induced inflammatory responses lead to the production of large amounts of pro-inflammatory cytokines, further increasing blood coagulability and viscosity, posing a risk of thrombosis and acute ischemic stroke [67,70]. Another potential mechanism of cerebrovascular complications is the “cytokine storm”, which is extremely intense in the case of patients with severe COVID-19 disease. As a consequence, C-reactive protein and other pro-inflammatory markers, including TNF-α, IL-6, and IL-8, are released in excess. Cytokine production is closely related to the functioning of the coagulation system, among others, by releasing tissue factor (TF), which is an activator of the extrinsic coagulation pathway. Moreover, pro-inflammatory factors contribute to the formation of neutrophil extracellular traps (NETs). These structures additionally activate coagulation pathways and stimulate the production of thrombin, leading to hypercoagulability [71,72]. Cytokine storm is a phenomenon that can dysregulate the immune response and damage the vascular endothelium and pulmonary alveolus, leading to internal organ failure and even death. This effect of SARS-CoV-2 infection may suggest that anti-inflammatory drugs may reduce the risk of serious complications of the disease [73,74].

Moreover, accumulating evidence suggests that cytokine storm coupled with oxidative stress contributes to COVID-19 pathogenesis and immunopathogenesis. This combination causes endothelitis and endothelial cell dysfunction. It also activates the blood clotting cascade, which results in blood coagulation and microvascular thrombosis. So, it is note-worthy that reactive oxygen species induced by SARS-CoV-2 infection can contribute to the development of ischemic stroke and thrombosis. Coronavirus’ infection of host cells results in an imbalance where ROS production rises while the host’s antioxidant responses decrease, culminating in increased redox stress. Oxidative stress can damage the endothelium of blood vessels, potentially contributing to the formation of clots, which can obstruct blood flow. Therefore, it appears that this mechanism may be a key contributor to the development of ischemic stroke and thrombosis, as it can interfere with normal blood flow and facilitate the formation of clots [75,76].

Severe COVID-19 has also been associated with elevated levels of D-dimers, fibrinogen, and activation of the coagulation cascade. Going further, platelets and coagulation factors participate in clot formation, which may lead to thrombosis and microvascular damage. Furthermore, immune system cells, such as macrophages, accumulate in the vascular walls and lead to the formation of atherosclerotic plaques. As a result, all of this may increase the risk of ischemic stroke [56,67,77]. On the contrary, some studies indicate that increased D-dimers levels are common in patients with viral infections and for that reason may not be necessarily associated with hypercoagulability [78,79]. Therefore, it is difficult to draw a single definitive conclusion confirming that hypercoagulability caused by SARS-CoV-2 infection may increase the risk of IS.

Additionally, recent studies suggest that the development of IS in patients with SARS-CoV-2 infection may potentially be related to the presence of anti-phospholipid antibodies (aPLs) in the serum. These mainly include: lupus anticoagulant (LAC), anti-cardiolipin antibodies (aCL), and anti-β2-Glycoprotein I antibodies (anti-β2-GPI) [78]. Beyrouti et al. examined six patients with COVID-19-related ischemic stroke and five of them were LAC-positive [79]. The study conducted by Capozzi et al. showed that 50% of studied patients tested positive for aCL IgG and IgG anti-β2-GPI was detected in 30% of patients [78]. Consequently, anti-phospholipid antibodies are suspected of playing a role in increasing blood clotting and activating pathways that lead to the production of pro-inflammatory cytokines in COVID-19 patients. Nevertheless, it should be taken into account that these antibodies are sometimes also found in healthy individuals, and only transiently during SARS-CoV-2 infection. Therefore, despite ongoing research, the exact mechanisms of the formation and action of circulating aPL remain unclear and require further observation [60,80,81].

**Figure 2:**
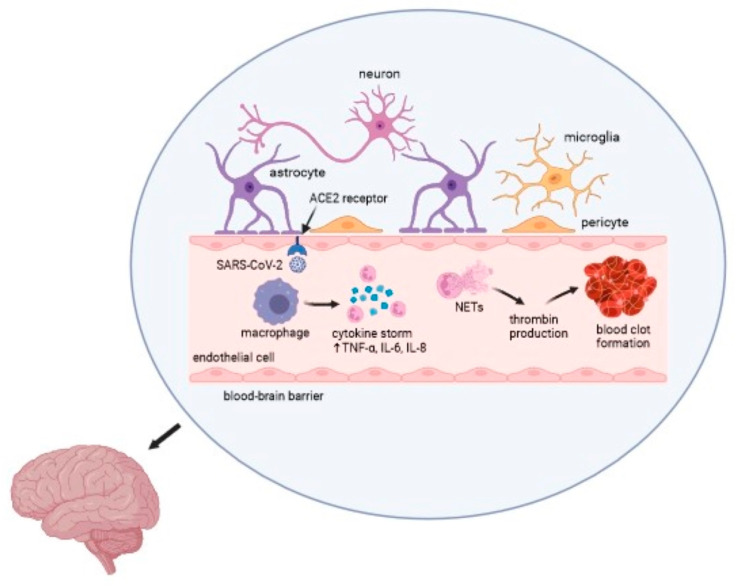
Mechanism of the SARS-CoV-2 infection in an ischemic stroke. Captions: ACE2—angiotensin converting enzyme 2; TNF-α—tumor necrosis factor α; IL—interleukin; NETs—neutrophil extracellular traps.

COVID-19 causes a wide range of clinical manifestations, including neurological disorders, thrombotic events, and other life-threatening complications. Multiple clinical studies have reported an ischemic stroke in patients with SARS-CoV-2 infection. The impact of viruses on the development of cerebrovascular events is undoubtedly significant. Nevertheless, further investigation is necessary to determine the precise etiology and pathomechanism of a stroke associated with COVID-19 [53,57,59]. The overall mechanism of action of the virus is presented in Figure 2.

## 4. Alzheimer’s Disease

Alzheimer’s disease is the leading cause of dementia worldwide, accounting for 50–70% of dementia cases. It is an irreversible neurodegenerative disease that significantly affects patients’ ability to function on a daily basis. Globally, Alzheimer’s disease incidence is expected to rise with aging populations and longer life expectancies, especially in developing countries [82]. Comprehensively, it has been estimated that approximately 50 million individuals suffer from dementia, and by 2050, this number is expected to triple [83]. AD affects both sexes to varying degrees. The global age-specific disease prevalence in women is 1.17 times higher than in men. Women also have a higher age-standardized death rate than males [84]. Alzheimer’s disease begins several decades before clinical symptoms appear [84]. The main features of patients with AD are elevated levels of amyloid-β (Aβ), which forms extracellular senile plaques, and hyperphosphorylated tau protein (p-tau), which aggregates intracellularly in the form of neurofibrillary tangles (NFTs). All of the above mentioned changes occur in brain tissue [85].

Alzheimer’s disease is a complex and multifactorial disease influenced by both genetic (hereditary) and environmental factors that affect the individual throughout life [84]. Environmental factors influencing Alzheimer’s disease include psychosocial factors including educational achievement, cognitive activity, bilingualism, social involvement, depression, and stress; pre-existing diseases such as diabetes, hypertension, dyslipidemia, obesity, cardiovascular disease, traumatic brain injury, hyperhomocysteinemia, hearing loss, and oral diseases; lifestyle, which includes physical activity, diet, sleep disorders, smoking, drinking alcohol, coffee and tea and other factors including environmental pollution as well as bacterial/viral infections—pathogens like *Chlamydia pneumoniae, Porphyromonas gingivalis*, *Salmonella typhimurium*, Hepatitis C virus, Human herpesvirus 5, 6A and 7, Herpes simplex virus 1 (HSV-1), Cytomegalovirus (CMV), Human immunodeficiency virus HIV-1, Human T cell leukemia virus type I (HTLV-1) [82,86,87,88]. A summary of the most common environmental pro- and anti-AD factors is shown in Table 1.

Alzheimer’s disease has been repetitively associated with viral etiology [89]. Two main pathways of viral involvement in the pathology of Alzheimer’s disease have been stated. The first is the direct way, where microorganisms directly infect the brain and promote the accumulation of Aβ and hyperphosphorylation of tau protein. The second is the indirect route, resulting from the inflammatory effects of the infection [90]. The recent pandemic caused by SARS-CoV-2 has provided evidence of viral involvement in Alzheimer’s disease [91].

Interestingly, it has been suggested that SARS-CoV-2 may infect the central nervous system. The virus can enter the CNS through several different routes. The first one is the bloodstream spread through infected leukocytes that reach the brain. Another mechanism is the entry of the virus with the use of the spike protein on its surface which binds to the ACE2 receptor on the BBB endothelial cells and via the blood-brain barrier arrives in glial cells and infects them [92,93,94]. Afterward, it passes through the synapses of infected neurons. It has been also assumed that SARS-CoV-2 may reach the CNS from the nasopharynx through the olfactory nerve to the olfactory bulb [93].

The study by Li et al. [95] highlights a significant and intriguing relationship between COVID-19 and an increased risk of AD. While COVID-19 is primarily a respiratory illness, its impact on the neurological system has become an area of growing concern and interest. The before mentioned study finds an association between COVID-19 and AD development. The research explores potential reasons, focusing on the severity of COVID-19, the role of immune-related pathways, the deterioration of lung health, and shared genetic factors. One of the key findings of the study was the suggestion that a severe course of COVID-19 may contribute to the development of Alzheimer’s disease, whereas mild cases of the virus infection do not seem to pose the risk of the same intensity. This is due to the fact that patients experiencing severe COVID-19 often undergo intense immune responses, which lead to systemic inflammation and damage across various organs, including the brain. Systemic inflammation may accelerate or trigger processes related to neurodegeneration, particularly in individuals who are predisposed to Alzheimer’s disease [95]. Moreover, a clear connection between lung health and cognitive function has been observed. Declining lung health in middle age has been associated with mild cognitive decline and an increased risk of dementia [85]. COVID-19 significantly impacts lung conditions, leading to inflammation and long-term respiratory issues [96]. This deterioration may, in turn, impair cognitive functions, exacerbating symptoms of Alzheimer’s disease. The study identifies 60 common genes expressed at high levels in the lungs, spleen, adipose tissue, and blood, shared by both COVID-19 and Alzheimer’s disease. These genes play a role in local immune responses in both conditions. Interestingly, in Alzheimer’s patients, the levels of these genes were additionally downregulated in the brain, likely due to disease-related changes. This genetic link suggests a common pathway that may increase susceptibility to Alzheimer’s disease when infected with COVID-19 [85]. Some researchers have also proposed that SARS-CoV-2 infection might lead to brain tissue atrophy, which is connected to damaging neurons and synapses, particularly around the brain’s ventricles [97]. The above mentioned findings provide valuable insights into the complex relationship between COVID-19 and Alzheimer’s disease. The unique association suggests that severe COVID-19 infections may trigger processes related to AD, primarily through immune-related pathways and the deterioration of lung health. The shared genetic factors and the potential for brain atrophy further underscore the intricate connection between these two conditions.

What is more, the study of Atkins J.L. et al. [98] found that pre-existing dementia was the main risk factor for severe SARS-CoV-2 infection. This factor was more significant than chronic obstructive pulmonary disease (COPD), type 2 diabetes, and depression [98]. This study revealed that the mortality rate due to COVID-19 infection among patients with dementia was higher than among those without dementia [98]. Alzheimer’s disease was the most common diagnosis of cognitive impairment among patients who died from COVID-19 according to a Spanish study conducted by Martín-Jiménez P. et al. [99]. Furthermore, COVID-19 survivors were shown to have a greater likelihood of being diagnosed with new-onset dementia within 6 months after infection than controls [100]. This indicates a mutual relationship in the occurrence of both diseases.

According to Atkins et al., SARS-CoV-2 infection in patients with dementia is distinguished by distinct clinical symptoms. The most common symptom is delirium, which occurs in 36.2% of all cases, while the control group has a frequency of 11.6%. Furthermore, other common COVID-19 symptoms, such as shortness of breath, muscle pain, chills, nausea, vomiting, and headache, were less frequently reported in individuals with dementia than in the control group [101]. According to a different study, at the onset of COVID-19, delirium and confusion developed in 82.4% of dementia patients, among which the most common cause of dementia was Alzheimer’s disease. Other common symptoms of COVID-19 included asthenia (76.8%), fever (72.8%), polypnea (51.2%), and desaturation (50.4%). Falls occurred in 35.2% of patients in the initial phase of the disease. A total of 19.2% of patients suffered from persistent disorientation and behavioral problems [102]. In a different study by Bianchetti A. et al. [103], the most common primary symptoms of SARS-CoV-2 infection were delirium and deterioration of functional status [103].

The ACE2/Ang-(1-7)/Mas axis plays an important role in maintaining normal cognitive functioning and protecting against neurodegeneration, as opposed to ACE and Ang II, which have been shown to impair cognitive functions [101,102,104,105]. The study conducted by Jiang T. et al. [106] discovered that Ang-1-7 levels were significantly reduced in the brain tissues of Alzheimer’s disease mice and that Ang-1-7 levels in the cerebral cortex and hippocampus were inversely related to tau hyperphosphorylation [107]. Another study, conducted by Liu S. et al. [108] found that patients with Alzheimer’s disease had reduced serum ACE2 activity than patients from control groups. A different study conducted by Ding Q. et al. [109] proved that Alzheimer’s patients’ brains had higher levels of ACE2 protein expression, regardless of age, gender, or disease severity [101].

Correspondingly, Aβ43 and Aβ42, longer forms of Aβ, are the main factors causing Aβ accumulation in the brain in the course of Alzheimer’s disease due to their neurotoxicity and high amyloidogenicity [110]. ACE2 has been found to convert Aβ43 to Aβ42, which is subsequently transformed by ACE to the less toxic Aβ40, which may have neuroprotective properties. This inhibits aggregation and delays the deposition of Aβ42 amyloid [111,112,113]. Aβ42 has been shown to have a high affinity for the S1 subunit of the SARS-CoV-2 virus spike protein and ACE2. In the study of Rudnicka-Drożak E. et al. [114] with artificially created SARS-CoV-2, Aβ42 was found to boost spike protein binding to ACE2, viral entry, and production of pro-inflammatory cytokine IL-6. In the case of Aβ40, no such effect was observed [114]. The study showed significantly increased ACE2 expression in the CA1 region of the hippocampus, temporal and occipital lobes. The temporal lobe and hippocampus are places particularly involved in the pathology of Alzheimer’s disease [115]. The results of this study suggest that SARS-CoV-2 infection of Alzheimer’s disease patients leads to increased virus infiltration into their brain cells when compared to healthy individuals, due to the increased expression of ACE2 in the course of AD [114]. ACE2 is an enzyme that regulates the release of brain-derived neurotrophic factor (BDNF), which plays an important role in neurodevelopment, neurogenesis, cognitive function, and prevention of neurodegeneration [44,45]. The study showed that a deficiency of this enzyme leads to impaired cognitive functions [116]. It can be suspected that SARS-CoV-2 infection, through inhibition of ACE2 and BDNF, may cause neurodegenerative changes by escalation of neuroinflammation, oxidative stress, and apoptosis [117].

Apolipoprotein E (ApoE) is a protein whose main function is the transport of cholesterol and other lipids to the neurons [118]. It is produced mainly in astrocytes and microglia cells, and under certain conditions also in neurons [116]. ApoE occurs in three isoforms that differ in their lipid transport properties and neuronal plasticity [118,119]. The study of Drouet B. et al. [120] showed that the occurrence of apoE2 and apoE3 isoforms is connected with the prevention of Aβ aggregation and neurotoxicity. These properties have not been demonstrated for apoE4 [120]. Individuals who carry the allele that encodes the apoE4 isoform have the highest risk of developing Alzheimer’s disease [121]. Data analysis performed by Kuo C.L. et al. [122] showed that apoE4 isoform homozygotes were more than two times more likely to test positive for COVID-19 than apoE3 isoform homozygotes. It was also discovered that the apoE4 isoform increases the risk of severe COVID-19, regardless of pre-existing dementia, cardiovascular diseases, or type 2 diabetes [122]. A different study showed that high blood cholesterol levels facilitate the entry of the SARS-CoV-2 virus into cells via ACE2 receptors by binding cholesterol to ApoE receptors [123]. These findings suggest a greater susceptibility of neurons and astrocytes encoded by the apoE4 isoform to SARS-CoV-2 infection and its subsequent increased severity [114].

The inflammation hypothesis in Alzheimer’s disease is one of the main theories about the pathology of the disease, along with Aβ deposition and the presence of tau tangles. Continuous activation of microglia and immune cells exacerbates the pathology of beta-amyloid plaques and tau proteins [124]. The brain of a patient suffering from Alzheimer’s disease is characterized by chronic inflammation. Activated microglial cells secrete pro-inflammatory cytokines such as interleukin-6 (IL-6), interleukin-1β (IL-1β), and tumor necrosis factor α (TNF-α) [125]. It was also found that in the case of AD patients, serum levels of IL-6 and TNF-α are higher compared to healthy individuals [126].

A state of excessive inflammation, the so-called “cytokine storm” is a characteristic feature of severe SARS-CoV-2 infection. Numerous pro-inflammatory cytokines were found to be elevated during COVID-19 [127]. This dysregulated immune response, resulting in elevated cytokine levels, may be due in part to activation of the NLR family pyrin domain-containing protein 3 (NLRP3) inflammasome by open reading frame 3a (ORF3a), an accessory protein of the SARS-CoV-2 virus [128,129,130,131]. Studies performed by Stancu et al. [132] and Heneka et al. [133] have shown that Aβ plaques and tau aggregates can stimulate the activation of the NLRP3 microglial inflammasome [132,133]. Activation of the NLRP3 inflammasome impairs normal microglial function, leading to reduced clearance of Aβ42 in the brain [134]. NLRP3 inflammasome activation has been implicated as a key neuroinflammatory pathway in Alzheimer’s disease, contributing to cognitive decline [135]. Since the ORF3a protein of the SARS-CoV-2 virus is able to activate the NLRP3 inflammasome, this mechanism may further enhance the already existing neuroinflammation caused by the activation of this inflammasome in the course of AD [136].

Oxidative stress has been found to be another important factor contributing to the initiation and progression of Alzheimer’s disease. Oxidative stress occurs as a result of redox imbalance due to excessive production of ROS, which ultimately leads to the loss of neurons [137]. Studies by Li et al. [138] have found that oxidative stress promotes the accumulation of Aβ. On the other hand, different studies by Matsouka et al. [139] have shown that Aβ promotes oxidative stress. A significant amount of evidence also suggests the involvement of oxidative stress in tau hyperphosphorylation and polymerization [140,141]. Moreover, numerous studies (i.e. by Hamilton et al. [142]) have shown that oxidative stress increases with age. In SARS-CoV-2 infection, oxidative stress has been found to play a role in perpetuating the cytokine storm and increased cellular hypoxia [143,144]. ROS are overproduced in response to SARS-CoV-2 infection as part of the toxic innate immune response against viral agents [145]. The addition of oxidative stress induced by ROS overproduction during SARS-CoV-2 infection to the already increased oxidative stress in AD patients due to age is a potential mechanism for disease intensification caused by COVID-19 [146].

**Figure 3:**
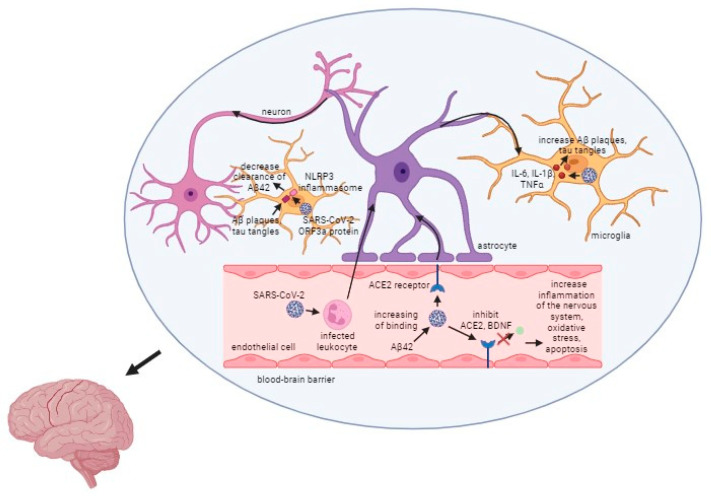
Mechanism of the SARS-CoV-2 infection in Alzheimer’s disease. Captions: Aβ/Aβ42—β amyloid plaques; ACE2—angiotensin converting enzyme 2; BDNF—brain-derived neurotrophic factor; NLRP3—NOD-, LRR- and pyrin domain-containing protein 3; ORF3a protein—open reading frame 3a protein.

The relationship between SARS-CoV-2, the virus responsible for COVID-19, and AD is a critical area of research. As one of the most common comorbidities associated with COVID-19, Alzheimer’s disease presents unique challenges and complications when co-occurring with this viral infection. One of the most significant impacts of the co-occurrence of Alzheimer’s disease and COVID-19 is the increased mortality risk among patients. Studies indicate that individuals with AD who contract COVID-19 have a much higher chance of experiencing severe outcomes, including death [114]. Several factors contribute to the elevated risk of severe COVID-19 infections in Alzheimer’s patients, including their age and vulnerability, as they are often older and have compromised health, as well as their weakened immune systems, which make it harder for them to fight off infections like SARS-CoV-2. Moreover, oxidative stress is a common factor in AD, contributing to neuronal damage. COVID-19 can exacerbate oxidative stress, worsening Alzheimer’s symptoms and potentially accelerating disease progression. The presence of the ApoE ε4 allele in some individuals is another link between COVID-19 and Alzheimer’s. This allele not only increases the risk of developing AD but also facilitates the entry of the SARS-CoV-2 virus into cells, leading to more severe infections. What is more, the angiotensin-converting enzyme 2 (ACE2) receptor is used by the SARS-CoV-2 virus to enter host cells. In Alzheimer’s disease, the expression of ACE2 is often increased, making these patients more susceptible to viral entry and infection [114]. While current data provide insights into how SARS-CoV-2 impacts Alzheimer’s disease, the exact mechanisms remain incompletely understood. More research is essential to fully unravel the interactions between these two conditions. The general mechanism of action of the virus is presented in Figure 3.

## 5. Conclusions

In summary, an association between COVID-19 and a number of neurodegenerative disorders has been observed. Alzheimer’s disease, ischemic stroke, and multiple sclerosis have all been linked to SARS-CoV-2 infection. The immunological pathways disrupted by COVID-19 significantly overlap with the pathogenic mechanisms of MS, IS, and AD. It suggests that SARS-CoV-2 infection could serve as an environmental risk factor for neurological disease manifestation in susceptible individuals. The SARS-CoV-2 virus causes activation of the NLRP3 inflammasome and production of interferons and neurotoxic cytokines that harm the myelin sheaths, which are responsible for protecting nerve cells. The body starts to synthesize autoantibodies that attack glial cells and neurons, harming the anatomical components of the brain which contributes to the onset of multiple sclerosis. Furthermore, the SARS-CoV-2 virus exacerbates the disease in individuals who are already suffering from MS by causing inflammation and nerve cell destruction through its influence on the synthesis of pro-inflammatory cytokines. Going forward, the release of pro-inflammatory cytokines may contribute to an increase in blood clotting. Cytokine storm affects the activation of coagulation pathways and the formation of NETs, leading to hypercoagulability. This condition can result in thrombosis, which blocks the flow of blood to the brain and causes an ischemic stroke. Severe inflammation and increased oxidative stress have been described in Alzheimer’s disease. Increased manifestation of these alterations has been observed during SARS-CoV-2 infection. It was observed that the SARS-CoV-2 virus enters cells more easily when the ACE2 receptor, whose expression is already elevated in AD, and the ApoE ε4 allele (which occurs in some individuals) are present. In this regard, the disease worsens and advances more quickly at the same time increasing the mortality rate of AD patients. Interestingly, mitochondrial dysfunction caused by SARS-CoV-2 infection leads to intracellular dysregulation and a decrease in ATP production by damage and mutations in mitochondrial DNA. This disrupts the transmission of electrical signals and leads to damage of neurons, contributing to the development of neurodegenerative diseases. The above findings indicate that there are several connections between the SARS-CoV-2 infection and the severity as well as incidence of selected neurodegenerative diseases. The described pathomechanisms may help to understand the impact of viral infection on the functioning of the nervous system. Despite that, COVID-19 is still a relatively new and little-understood disease. For this reason, it is difficult to determine its long-term effects and the impact on the development of severe complications, which constitutes some limitations of the study. New studies on this issue are still being published and more accurate information may appear in the future. Hence, more extensive research on that topic needs to be conducted to clearly determine the impact of SARS-CoV-2 infection on the development of neurological diseases. A good direction for future research would be to clearly determine how the virus affects the development of neurodegenerative diseases, whether there are certain groups of patients in whom contact with the virus poses a greater risk of developing and intensifying disease symptoms, and how it would be possible to prevent the development of diseases.

## Figures and Tables

**Table 1:** The influence of the most popular environmental factors contributing to the increased incidence of Alzheimer’s disease.

	Anti-AD Factors	Pro-AD Factors
psychosocial factors	educational achievement, cognitive activity, bilingualism, social involvement	depression, stress
pre-existing diseases		diabetes, hypertension, dyslipidemia, obesity, cardiovascular disease, traumatic brain injury, hyperhomocysteinemia, hearing loss, oral diseases
lifestyle	physical activity, Mediterranean diet, DASH diet, MIND diet, coffee and tea drinking	sleep disorders, smoking, drinking alcohol
other factors		environmental pollution, pathogens
